# A comparison of utilization and short-term complications of technology-assisted versus conventional total knee arthroplasty

**DOI:** 10.1186/s43019-022-00143-5

**Published:** 2022-03-18

**Authors:** Trevor Simcox, Vivek Singh, Christian T. Oakley, Omid S. Barzideh, Ran Schwarzkopf, Joshua C. Rozell

**Affiliations:** 1 Department of Orthopedic Surgery, NYU Langone Hospital – Long Island, Mineola, NY USA; 2 Department of Orthopedic Surgery, NYU Langone Health, New York, NY USA; 3 Division of Adult Reconstruction, Department of Orthopedic Surgery, NYU Langone Orthopedic Hospital, Hospital of Joint Diseases, 301 East 17th Street, New York, NY 10003 USA

**Keywords:** Computer-assisted, Technology, Robotics, Total knee arthroplasty, Navigation

## Abstract

**Background:**

While technology-assisted total knee arthroplasty (TA-TKA) improves implant positioning, whether it confers improved clinical outcomes remains inconclusive. We sought to examine national TA-TKA utilization trends and to compare outcomes between TA-TKA and unassisted TKA (U-TKA).

**Methods:**

Patients who underwent primary, elective TKA from 2010 to 2018 were identified using the American College of Surgeons National Surgical Quality Improvement Program (NSQIP) database. Demographic, perioperative, and 30-day outcomes were collected. Patients were stratified on the basis of whether they underwent TA-TKA, which included computer navigation and robotics, or U-TKA. The proportion of patients undergoing TKA using TA-TKA was calculated. One-to-one propensity-score matching paired patients undergoing TA-TKA or U-TKA. Independent samples *t*-tests and Mann–Whitney *U* tests were used to compare continuous variables, and chi-squared tests were used to compare categorical variables.

**Results:**

Of the 402,284 TKA patients, 10,429 (2.6%) cases were performed using TA-TKA. Comparing the unmatched TA-TKA and U-TKA groups, race (*p* < 0.001), smoking status (*p* = 0.050), baseline functional status (*p* < 0.001), and body mass index (BMI) (*p* < 0.001) significantly differed. Propensity-score matching yielded 8633 TA-TKA and U-TKA pairs. The TA-TKA cohort had shorter hospital length of stay (LOS) (2.7 ± 2.5 versus 2.8 ± 1.9 days, *p* = 0.017) but similar operative times (92.4 ± 33.4 versus 92.6 ± 39.8 min, *p* = 0.670). Compared with the U-TKA group, the TA-TKA group had lower major complication (7.6% versus 9.4%, *p* < 0.001) and transfusion (3.9% versus 5.1%, *p* < 0.001) rates and higher rates of discharge to home (73.9% versus 70.4%, *p* < 0.001). Reoperation and readmission rates did not significantly differ between groups.

**Conclusions:**

TA-TKA utilization remains low among orthopedic surgeons. Compared with U-TKA, TA-TKA yielded improved perioperative and 30-day outcomes. Nonetheless, surgeons must consider the benefits and drawbacks of TA-TKA when determining the proper surgical technique and technology for each patient.

**Level III evidence:**

Retrospective cohort study.

## Background

Over one million cases of total knee arthroplasty (TKA) are performed in the USA every year [1]. It is one of the most successful elective operations in terms of patient satisfaction and improved quality of life [2]. With the introduction of technology-assisted total knee arthroplasty (TA-TKA), orthopedic surgeons may now achieve increased accuracy and precision in the placement of TKA components [3, 4]. Despite this, there is no consensus on whether TA-TKA confers superior clinical outcomes as compared with conventional unassisted techniques [5–10]. Therefore, larger and more comprehensive studies are required to detect meaningful clinical benefits of using advanced technologies.

The term TA-TKA encompasses both robotic and computer-navigated TKA and relies on a combination of handheld sensors, robotic arms, imaging, and/or specialized navigation cameras to detect the anatomic and mechanical alignment of the knee. Computer navigation provides surgeons with intraoperative real-time positioning information, but does not actively perform bone resection. Navigation may be further categorized into image-based or imageless systems depending on if preoperative imaging is utilized within the navigation system to determine implant orientation. Robotic systems, on the other hand, may offer three-dimensional intraoperative data and aid in performing bony resection. Currently, some robotic systems utilized in clinical practice rely on preoperative imaging to identify patient anatomy and to construct a plan. Robotic systems are categorized into active and semi-active systems depending on whether the robot performs bone preparation independently or in conjunction with the operator [11].

Although TA-TKA has been available for several decades, TA-TKA has not been universally adopted [12] and accounts for only 5–10% of TKAs performed in the USA [13, 14]. This low utilization rate may be attributable to costly capital investment for hospital systems, increased operative times, a significant learning curve alongside training burden, and skepticism from operators who have already achieved quality outcomes utilizing unassisted techniques [15–17]. Moreover, institutions must also train support staff and optimize operating room workflow before realizing the full potential of TA-TKA [18].

Technology utilization rates may vary across geographic regions, hospital systems, and surgeons. In one recent analysis of computer navigation TKA trends within the USA, Western hospitals were more likely to employ navigation as compared with Midwestern and Southern institutions [19]. Moreover, the authors found that technology utilization increased from 1.2% to 7.0% of cases between 2005 and 2014, a trend supported by additional analyses [16, 20]. Further assessment of utilization trends will help elucidate how these technologies are being implemented on the national level.

The purpose of this study is to investigate the national trends in the utilization of TA-TKA using the American College of Surgeons National Surgical Quality Improvement Program (NSQIP) Database, to compare short-term major complication, readmission, and reoperation rates, and to examine differences in hospitalization and discharge trends between patients undergoing unassisted and TA-TKA.

## Methods

### Database

The NSQIP database was queried from 2010 to 2018, the most recent year with released data at the time of data analysis. The database itself did not contain any TKA operations performed using navigation technology prior to 2010. It contains detailed perioperative and 30-day postoperative complication data collected from patients undergoing surgery at one of approximately 700 participating hospitals in the USA. Data were prospectively collected and clinically verified by trained reviewers at each institution [21]. Because the NSQIP data are de-identified, this study was exempt from institutional review board (IRB) approval at our institution.

### Generation of study groups

Current Procedural Terminology (CPT) codes and International Classification of Diseases (ICD) Ninth and Tenth Revision Diagnosis Codes were used for identifying patient groups. The CPT code 27,447 was used to identify patients over 18 years who underwent TKA within the NSQIP database. Patients undergoing TKA for non-elective indications such as trauma, malignancy, revision procedure, or infection and had ICD-9/ICD-10 codes reflective of these indications were excluded. Additionally, cases missing relevant perioperative or operative data were excluded from the analysis. Patients were assigned to either TA-TKA or U-TKA study groups on the basis of the presence of a secondary CPT code: 20985, 0054 T, or 0055 T. The code 0054 T refers to TKA using fluoroscopically guided navigation, 0055 T refers to TKA using computed tomography (CT)/magnetic resonance imaging (MRI) to reconstruct the three-dimensional anatomy of the joint, and 20985 refers to all navigation systems that do not use imaging for registration of the anatomic and mechanical axes of the limb.

### Variables studied

The primary outcomes were major complications within 30 days following surgery. We defined a major complication as any of the following: cardiac arrest, myocardial infarction, cerebrovascular accident, wound dehiscence, respiratory failure with inability to wean from ventilator, renal failure, deep organ-space infection, postoperative transfusion, deep vein thrombosis (DVT), pulmonary embolism (PE), pneumonia, postoperative reintubation, periprosthetic wound infection (deep wound infection), sepsis and septic shock, reoperation, and death. Major complications were then categorized on the basis of type of postoperative complication. Readmission was not listed as a postoperative complication. Secondary outcomes were operative time, hospital length of stay (LOS), and discharge destination. Operative time was defined as the time difference from the initial skin incision to skin closure. LOS was defined as the number of postoperative days a patient was admitted to the hospital. Discharge destinations were categorized into home, unskilled facility, rehab/skilled nursing facility, expired/hospice, and other. Each readmission and reoperation diagnosis code was manually reviewed and categorized for subgroup analysis. Patient baseline demographic information and comorbidities, including year of operation, ethnicity/race, age, American Society of Anesthesiologists’ (ASA) classification, functional status, body mass index (BMI; kg/m^2^), and five-factor modified frailty index (mFI) score, were collected.

### Statistical analysis

All analyses were performed using SPSS v25 (IBM Corporation, Armonk, New York). Patient baseline demographics and comorbidities were first compared between cohorts. To account for all baseline characteristics, propensity score matching was used [22]. A 1:1 match was performed using a balanced, nearest-neighbor propensity score [23]. This method of cohort matching has been established by previous literature as an optimal method for estimating differences between treatment groups [24]. Post-matching analysis was performed to confirm the quality of matching between TA-TKA and U-TKA groups. All primary and secondary outcome variables were assessed between matched cohorts.

A *p*-value of less than 0.05 was considered statistically significant. Chi-squared, independent sample two-sided *t*-test or Mann–Whitney *U* test was used to find differences between study groups based on the variable type and whether the data were normally distributed. The Bonferroni correction for *p*-value was used when performing multiple-comparison chi-squared analysis. All descriptive data are represented as mean ± standard deviation.

## Results

**Fig. 1:**
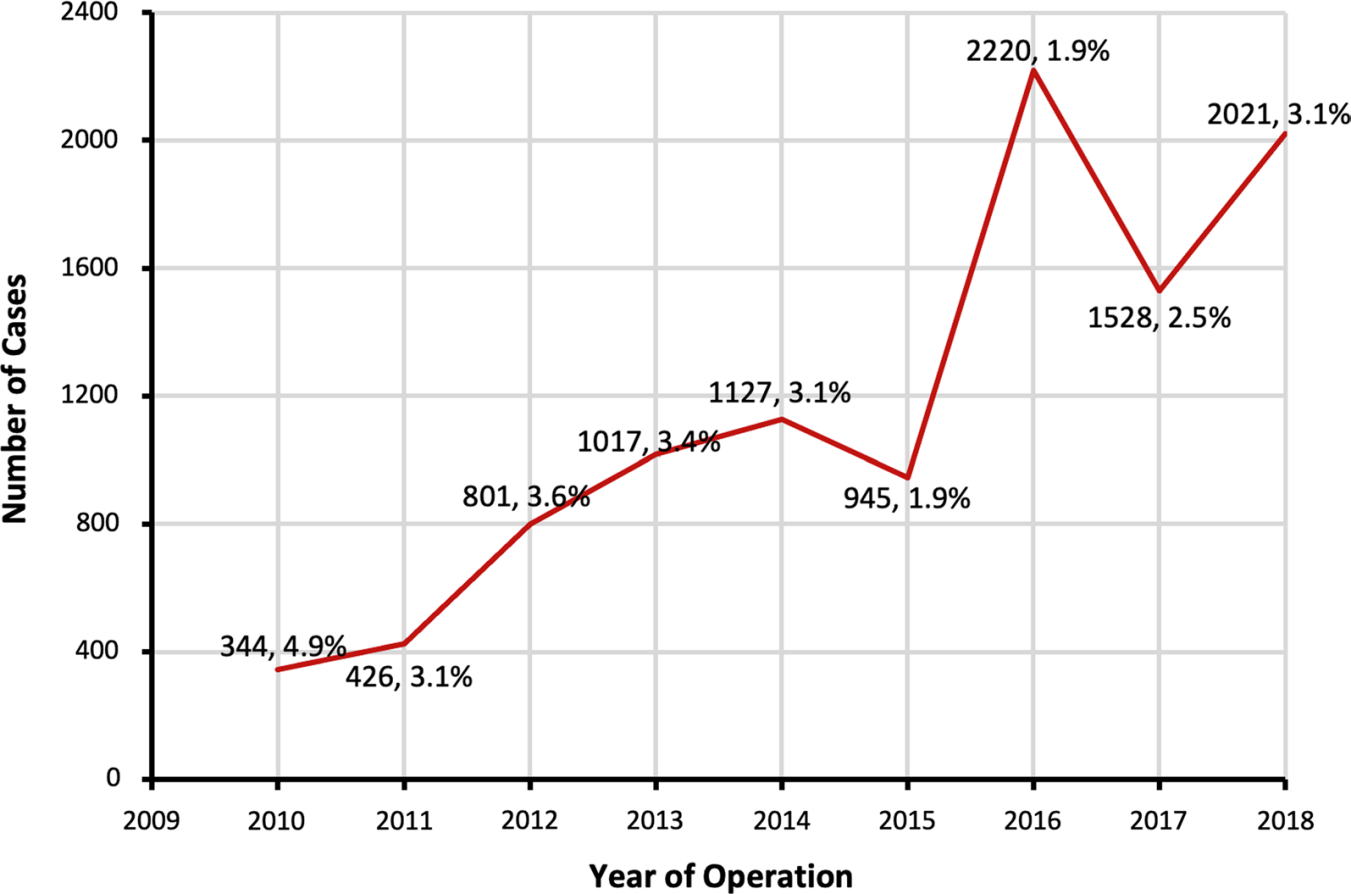
Case volume of technology-assisted TKA by year within the NSQIP database. The yearly listed percentages equal the number TA-TKA performed divided by the total number of TKAs performed during that respective calendar year. The proportion of patients undergoing TA-TKA per year decreased during the study period (*p* < 0.001)

In total, 402,284 primary TKA patients met inclusion criteria, of which 10,429 (2.6%) cases were performed using intraoperative technology and assigned to the TA-TKA cohort. Within the TA-TKA cohort, 10,100 (97%) cases were coded with the CPT 20985, 144 (1.4%) as 0054T, and 185 (1.8%) as 0055T. The proportion of TKAs performed using technology decreased during the study period (slope = 0.1%/year, *p* < 0.001, *R*2 = 0.000) (Fig. 1). Upon propensity score matching, 8663 patients in the U-TKA were successfully matched to 8663 in the TA-TKA group.

Baseline characteristics were compared between unmatched TA-TKA and U-TKA study groups, and significant differences were found between groups (Table 1). Race distribution significantly differed between study groups (*p* < 0.001); white patients accounted for a disproportionately high percentage of TA-TKA performed compared with other races. In the TA-TKA cohort, fewer patients were smokers (7.8% versus 8.3%, *p* = 0.050). Baseline functional status differed between study groups (*p* < 0.001); there were fewer “partially dependent” (0.8% versus 1.2%, *p* < 0.001) and more “independent” (99.1% versus 98.3%, *p* < 0.001) patients in the TA-TKA cohort. ASA classification significantly varied between groups (*p* < 0.001), and TA-TKA patients were more likely to have severe disturbances and less likely to have no disturbances as compared with U-TKA. The TA-TKA cohort had lower mean BMI scores (32.6 ± 6.98 versus 32.9 ± 7.10), *p* < 0.001) and mFI scores (0.043 ± 0.087 versus 0.046 ± 0.091, *p* < 0.001) than those undergoing U-TKA. Sex and age category did not vary between groups. After propensity score matching, there was only one significant difference in baseline characteristics remaining between study groups; more patients undergoing TA-TKA had ASA classification scores of 4 as compared with U-TKA (0.9% versus 0.7%, *p* = 0.034).

**Table 1:** Baseline characteristics of unmatched and matched patient cohorts

Patient characteristic	Unmatched cohorts			Matched cohorts		
	U-TKA (*n* = 391,855)	TA-TKA (*n* = 10,429)	*p*-Value	U-TKA (*n* = 8633)	TA-TKA (*n* = 8633)	*p*-Value
Sex—no. (%)			0.732			0.826
Male	150,190 (38.3)	4009 (38.5)		3224 (37.3)	3239 (37.5)	
Female	241,547 (61.7)	6401 (61.5)		5409 (62.7)	5394 (62.5)	
Age category—no. (%)			0.428			0.905
18–49	13,580 (3.5)	339 (3.3)	0.236	143 (1.7)	157 (1.8)	0.415
50–59	73,640 (18.8)	1950 (18.7)	0.807	1516 (17.6)	1509 (17.5)	0.889
60–69	150,945 (38.5)	3971 (38.1)	0.358	3496 (40.5)	3460 (40.1)	0.576
70–79	117,523 (30.0)	3184 (30.5)	0.236	2767 (32.1)	2764 (32.0)	0.961
80–89	34,942 (8.9)	948 (9.1)	0.541	706 (8.2)	738 (8.5)	0.379
90+	988 (0.3)	33 (0.3)	0.198	5 (0.1)	5 (0.1)	1.000
Race—no. (%)			** < 0.001**			0.844
White	277,735 (70.9)	8016 (76.9)	** < 0.001**	7190 (83.3)	7226 (83.7)	0.461
Hispanic	19,923 (5.1)	537 (5.1)	0.766	272 (3.2)	281 (3.3)	0.697
Native American or Pacific Islander	3201 (0.8)	44 (0.4)	** < 0.001**	15 (0.2)	10 (0.1)	0.317
Black/African American	29,403 (7.5)	582 (5.6)	** < 0.001**	343 (4.0)	336 (3.9)	0.784
Asian	8164 (2.1)	133 (1.3)	** < 0.001**	67 (0.8)	62 (0.7)	0.659
Unknown	53,429 (13.6)	1117 (10.7)	n/a	746 (8.6)	718 (8.3%)	n/a
Smoking status (within 1 year)—no. (%)			**0.050**			0.801
Yes	32,501 (8.3)	809 (7.8)		410 (4.7)	402 (4.7)	
No	359,354 (91.7)	9620 (92.2)		8223 (95.3)	8231 (95.3)	
Functional status—no. (%)			** < 0.001**			1.000
Independent	385,003 (98.3)	10,340 (99.1)	** < 0.001**	8619 (99.8)	8619 (99.8)	1.000
Partially dependent	4601 (1.2)	80 (0.8)	** < 0.001**	14 (0.2)	14 (0.2)	1.000
Totally dependent	160 (0.0)	3 (0.0)	0.546	0 (0.0)	0 (0.0)	n/a
Unknown	2091 (0.5)	6 (0.1)	n/a	0 (0.0)	0 (0.0)	n/a
ASA classification—no. (%)			** < 0.001**			0.108
1—No disturbance	7518 (1.9)	134 (1.3)	** < 0.001**	80 (0.9)	78 (0.9)	0.873
2—Mild disturbance	190,918 (48.7)	5053 (48.5)	0.586	4460 (51.7)	4394 (50.9)	0.315
3—Severe disturbance	186,442 (47.6)	5045 (48.4)	0.108	4011 (46.5)	4104 (47.5)	0.156
4+—Life-threatening disturbance or moribund	6532 (1.7)	180 (1.7)	0.241	82 (0.9)	57 (0.7)	**0.033**
Non-assigned	445 (0.1)	17 (0.2)	n/a	0 (0.0)	0 (0.0)	n/a
BMI—mean (SD)	32.93 (7.10)	32.61 (6.98)	** < 0.001**	32.34 (6.25)	32.36 (6.32)	0.815
mFl score—mean (SD)	0.046 (0.091)	0.043 (0.087)	** < 0.001**	0.035 (0.078)	0.036 (0.079)	0.270

Bold values represent statistically significant differences (*p* < 0.05)

BMI, body mass index; mFl, five-factor modified frailty index; no., number; SD, standard deviation; TA-TKA, technology-assisted total knee arthroplasty; U-TKA, unassisted total knee arthroplasty

**Fig. 2:**
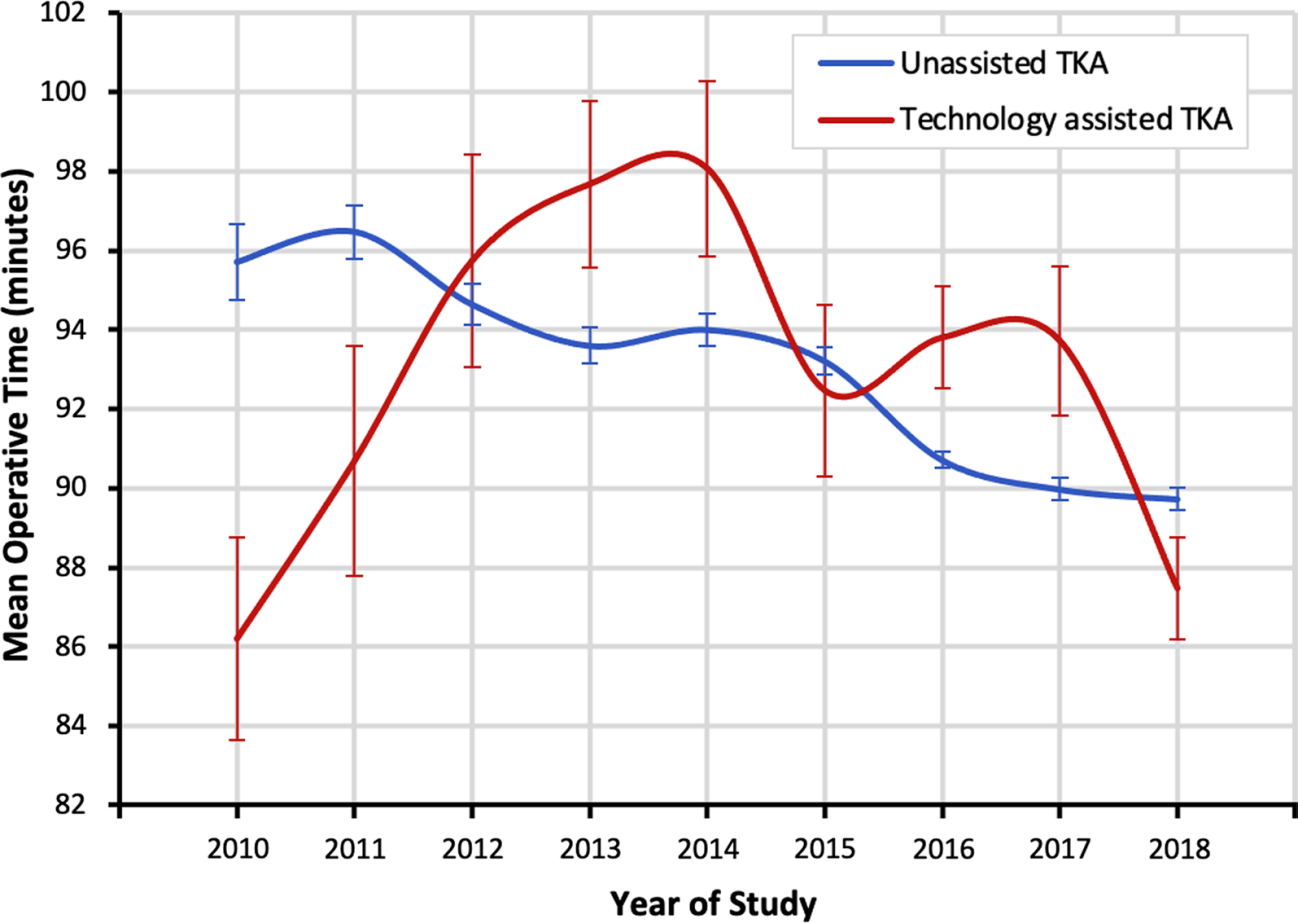
Mean operative time by year of operation for the unmatched TA-TKA and U-TKA cohorts. The mean operative time was significantly greater for the TA-TKA cohort as compared with U-TKA cohort (93.1 ± 33.7 versus 91.7 ± 36.9 min, *p* < 0.001). For both technology-assisted and unassisted cohorts, there was a negative correlation between operative year and operative time across the study period

Operative times were assessed for the unmatched and matched study groups (Fig. 2, Table 2). For the unmatched cohorts, the mean operative time for the TA-TKA cohort was significantly longer than that of the U-TKA cohort (93.1 ± 33.7 versus 91.7 ± 36.9 min, *p* < 0.001). After propensity score matching, the mean operative times for TA-TKA and U-TKA were similar (92.6 ± 39.8 versus 92.4 ± 33.4 min, *p* = 0.670). There was a significant decrease in operative time for the U-TKA (slope = −0.940 min/year, *p* < 0.001, *R*2 = 0.003) and TA-TKA (slope = −0.640 min/year, *p* < 0.001, *R*2 = 0.002) cohorts across the years of study. In a time series analysis of the TA-TKA cohort, operative times increased from 2010 to 2013 (slope = 3.747 min/year, *p* < 0.001, *R*2 = 0.013) and decreased from 2014 to 2018 (slope = −2.139 min/year, *p* < 0.001, *R*2 = 0.007).

**Table 2:** Comparison of perioperative variables between matched unassisted and technology-assisted cohorts

	U-TKA (*n* = 8633)	TA-TKA (*n* = 8633)	*p*-Value
Operative time (min)—mean (SD)	92.6 (39.8)	92.4 (33.4)	0.670
Length of stay (days)—mean (SD)	2.76 (1.86)	2.68 (2.51)	**0.017**
Discharge destination—no. (%)			** < 0.001**
Home	6077 (70.4)	6376 (73.9)	** < 0.001**
Unskilled facility	7 (0.1)	4 (0.1)	0.366
Rehab/skilled nursing facility	2238 (25.9)	1964 (22.7)	** < 0.001**
Expired/hospice	9 (0.1)	2 (0.0)	**0.035**
Other/AMA	16 (0.2)	6 (0.1)	**0.033**
Unknown	286 (3.3)	281 (3.3)	0.831
Major complication—no. (%)	810 (9.4)	659 (7.6)	** < 0.001**
Any wound complication	24 (0.3)	25 (0.3)	1.000
Periprosthetic infection	5 (0.1)	6 (0.1)	0.774
Transfusion	439 (5.1)	336 (3.9)	** < 0.001**
Systemic infection	24 (0.3)	23 (0.3)	1.000
DVT/PE	105 (1.2)	98 (1.1)	0.672
Cardiac	19 (0.2)	15 (0.2)	0.607
Respiratory	76 (0.9)	59 (0.7)	0.167
Neurologic	8 (0.1)	5 (0.1)	0.581
Renal	6 (0.1)	1 (0.0)	0.125
Reoperations—no. (%)	75 (0.9)	87 (1.0)	0.344
Unrelated operation	31 (0.4)	31 (0.4)	1.000
Periprosthetic fracture	8 (0.1)	5 (0.1)	0.405
Superficial irrigation and debridement	7 (0.1)	12 (0.1)	0.251
Periprosthetic infection	10 (0.1)	14 (0.2)	0.414
Hardware complication or dislocation	10 (0.1)	8 (0.1)	0.637
Other operation related to TKA	9 (0.1)	17 (0.2)	0.116
Readmission—no. (%)	275 (3.2)	236 (2.7)	0.080
Unspecified	58 (0.7)	63 (0.7)	0.648
Gastrointestinal	23 (0.3)	24 (0.3)	0.884
Cardiac	23 (0.3)	15 (0.2)	0.194
Pulmonary	15 (0.2)	14 (0.2)	0.853
Hematological	35 (0.4)	36 (0.4)	0.905
Other medical diagnosis	37 (0.4)	22 (0.3)	**0.050**
Unrelated musculoskeletal diagnosis	16 (0.2)	9 (0.1)	0.161
Periprosthetic fracture	4 (0.0)	4 (0.0)	1.000
Superficial surgical site infection	46 (0.5)	27 (0.3)	**0.026**
Periprosthetic infection	14 (0.2)	18 (0.2)	0.479
Unspecified TKA complication or dislocation	4 (0.0)	4 (0.0)	1.000
Mortality—no. (%)	4 (0.0)	9 (0.1)	0.267

Bold values represent statistically significant differences (*p* < 0.05)

No., number; SD, standard deviation; TA-TKA, technology-assisted total knee arthroplasty; U-TKA, unassisted total knee arthroplasty

Inpatient hospitalization variables were also assessed between matched cohorts. Patients undergoing TA-TKA had significantly shorter mean LOS compared with U-TKA (2.68 ± 2.51 versus 2.76 ± 1.86 days, *p* = 0.017). The discharge destination of the matched cohorts differed as well (*p* < 0.001). In the TA-TKA cohort, more patients were discharged home (73.9% versus 70.4%, *p* < 0.001) and fewer were discharged to rehab/skilled nursing facilities (22.7% versus 25.9%, *p* < 0.001).

The major complication rate in the TA-TKA cohort was significantly lower than the U-TKA cohort (7.6% versus 9.4%, *p* < 0.001). In subgroup analysis of major complication rates, fewer patients received blood transfusions in the TA-TKA cohort as compared with U-TKA cohort (3.9% versus 5.1%, *p* < 0.001). There were no significant differences in wound (*p* = 1.000), periprosthetic infection (*p* = 0.774), systemic infection (*p* = 1.000), DVT or PE (*p* = 0.672), cardiac (*p* = 0.607), respiratory (*p* = 0.167), neurologic (*p* = 0.581), or renal (*p* = 0.125) complications. Although readmission rates between TA-TKA and U-TKA did not significantly differ (2.7% versus 3.2%, *p* = 0.080), the TA-TKA cohort trended toward lower readmission rates. TA-TKA patients were less likely to be readmitted for other medical diagnoses (0.3% versus 0.4%, *p* = 0.050) and superficial surgical site infection (0.3% versus 0.5%, *p* = 0.026). On subgroup analysis, there were no significant differences in the number of patients readmitted for periprosthetic fracture (*p* = 1.000), periprosthetic infection (*p* = 0.479), or unspecified TKA complications and dislocations (*p* = 1.000).

Reoperation (1.0% versus 0.9%, *p* = 0.344) and mortality (0.1% versus 0.0%, *p* = 0.267) rates did not significantly differ between TA-TKA and U-TKA cohorts. On subgroup analysis, there were no significant differences in the proportion of patients undergoing superficial irrigation and debridement for superficial infections (*p* = 0.251), revision for periprosthetic infections (*p* = 0.414), periprosthetic fracture (*p* = 0.405), hardware complications or dislocations (*p* = 0.637), or other operations related to TKA (*p* = 0.116). Additionally, there was no significant difference in the number of patients undergoing unrelated operations (*p* = 1.000).

## Discussion

The use of technology in TKA may allow surgeons to achieve more accurate and precise implant placement. However, whether this translates to improved clinical outcomes, if certain patient populations benefit from intraoperative technology over unassisted techniques, or if advanced technologies confer improved long-term implant survivorship remains an area of active research.

Our findings generally support prior literature findings, which demonstrated that TA-TKA has not been universally adopted in the USA [16, 19, 20, 25]. We found that only 3.1% of patients underwent primary TKA with the use of intraoperative technology in the year 2018. In contrast, a National Inpatient Sample (NIS) database study found utilization rates for primary TKA to be 7.0% in 2014, a rate which has likely further increased in recent years [19]. Notably, the NSQIP and NIS databases include data from different subsets of hospitals. Geographic region and socioeconomic status influence TA-TKA utilization rates, whereas TA-TKA is more likely to be performed at high-volume, urban, and teaching hospitals [16, 19]. Furthermore, patients with private insurance are more likely to undergo surgery with technology as opposed to conventional methods [16, 26]. Another NSQIP database study conducted between 2012 and 2018 found that 2.6% of primary TKAs were performed with the assistance of computer navigation [25]. On the other hand, in an analysis of the New York Statewide Planning and Research Cooperative System (SPARCS) database, robotic TKA utilization rates were lower than 1.5% [19, 20]. Although database studies are well suited to determine national trends, they are limited by the number of hospitals that participate in data collection and often take multiple years to release datasets. Since the last year included in this study, we would expect the utilization rates of TA-TKA to have increased each year.

Surprisingly, the annual proportion of TKAs performed using technology within the NSQIP database decreased from 2010 to 2018. A recent study found that the utilization of computer-assisted TKA increased from 4.9% to 9.5% in New York and from 4.0% to 5.7% in Florida between the years 2010 and 2017 [14]. An NIS database analysis found that the proportion of TA-TKA increased steadily from 1.2% in 2005 to 7.0% in 2014 [19]. Our contradictory results may be explained by the increase in the number of participating centers in the NSQIP database during the study period, which coincides with the decrease in technology use. These additional centers may be performing TA-TKA at lower rates than the NSQIP-participating institutions at the start of the study period.

We found significant differences in race between the unmatched TA-TKA and U-TKA groups. In the TA-TKA cohort as compared with the U-TKA cohort, Asian, Black/African Americans, and Native American/Pacific Islanders were underrepresented and Whites overrepresented. This highlights the potential for racial disparities in TA-TKA utilization across the US health system. In an NIS database analysis, African American patients were more likely to undergo TA-TKA as compared with Whites, Hispanics, Asians or Pacific Islanders, and Native Americans [19]. Several authors have suggested that patient income, geographic region, and socioeconomic status may significantly influence the probability of receiving TA-TKA [16, 19]. Since the NSQIP database lacks hospital and geographic data, we were unable to investigate these geographic or socioeconomic trends.

To our knowledge, this is the largest study to compare operative times for patients undergoing TA-TKA and U-TKA. Prior studies have shown that TA-TKA and U-TKA have similar operative times, and not surprisingly, we found that mean operative times did not significantly differ between the matched TA-TKA and U-TKA cohorts [8, 10, 25, 27, 28]. Although these data support existing findings, operators using technology must register bony landmarks, interpret intraoperative data, and adjust component positions accordingly. These additional steps have the potential to increase operative time, but high-volume adult reconstructive knee surgeons may perform these swiftly such that no clinically meaningful increase in operative time is observed. During the study period, TA-TKA operative times decreased, suggesting that hospital systems and surgeons have become more efficient over time as they emerged from their initial learning curve and as the technology itself has substantially improved over time. Notably, operative times in the TA-TKA cohort increased in the first few study years before decreasing by a greater amount in the latter study years. The number of participating centers in the NSQIP database increased during the first few years, and the increased operative time may be due to these additional centers reporting patients with longer procedure times.

In examining inpatient outcomes, we found that patients undergoing TA-TKA had significantly shorter LOS as compared with that of U-TKA. In the existing literature, the association between TA-TKA and LOS is unclear. Several studies have found either similar [27, 28] or longer LOS [8, 29] in patients undergoing TA-TKA, but our analysis is one of the first to report shorter LOS. Additionally, patients in the TA-TKA cohort were more likely to be discharged home and less likely to be discharged to rehabilitation or skilled nursing facilities as compared with the U-TKA cohort. Since the NSQIP database lacks hospital data, we were unable to explain these trends, though varying institutional discharge policies across individual hospital systems may account for these differences. It is important to note that institutions supporting robotic and navigation technology may also have more robust perioperative protocols, same-day surgery programs, and physical therapy resources, possibly leading to improved in-patient outcomes as well.

Patients undergoing TA-TKA had lower complication rates than those undergoing U-TKA. A recent meta-analysis comparing robotic and unassisted TKA observed similar complication rates between groups [30], though some analyses comparing computer-assisted and unassisted TKA have demonstrated lower complication rates in those undergoing computer-assisted TKA [8, 13]. In our study, TA-TKA patients had significantly lower postoperative transfusion rates compared with U-TKA patients, which is consistent with prior findings [8, 13, 28] and associated with improved surgical outcomes [31]. Postoperative readmission rates did not significantly differ between groups. In prior national database analyses, computer-assisted TKA has yielded similar [25] and decreased [13] readmission rates. Further analysis revealed that patients in the TA-TKA cohort were readmitted primarily for superficial surgical site infections and hematological issues in addition to unspecified and other medical diagnoses. Manually reviewing the diagnosis codes of readmitted patients, it was difficult to determine whether certain readmissions were caused by periprosthetic fracture or hardware complications as well, possibly causing under- or overreporting of these conditions in our analysis. Nonetheless, the trend toward lower readmission rates for TA-TKA patients may be due to reduced need for bone and periarticular soft tissue manipulation intraoperatively due to improved component positioning and tracking [32]. Moreover, fewer TA-TKA patients were discharged to skilled nursing facilities, which is associated with increased readmission rates as well [33]. Lastly, reoperation and mortality rates did not differ between groups, which is consistent with previous findings [27, 28].

Surgeons who perform TA-TKA and U-TKA may work in different regions of the country or different practice environments; this provides one possible explanation for the perioperative differences observed between TA-TKA and U-TKA groups. Prior studies assessing TA-TKA utilization have demonstrated that it is more likely to be performed in urban, teaching, and high-volume hospitals [16, 19]. Given this, we hypothesize that TA-TKA cases in the NSQIP database were predominantly performed by operators at urban, tertiary referral centers, orthopedic specialty hospitals, or ambulatory surgery centers. This may explain the observed differences in perioperative outcomes.

For certain complications, our analysis produced slightly different incidence rates on subgroup analysis of major complications, readmissions, and reoperations. This discrepancy is due to coding differences within the database for each category of complication. Since the standard of care for a deep hardware infection involves operative management, we believe that infection rates are best approximated in the reoperation subgroup analysis.The NSQIP database lacks the granularity in ICD diagnosis coding needed to capture orthopedic-specific complication rates, and therefore, the reoperation subgroup analysis provides the most accurate measure of infection rates. Additionally, the NSQIP database does not record complications treated on an outpatient basis or through emergency department care, and this causes underreporting of certain postoperative complications—notably superficial wound complications, suboptimal functional outcomes, and other low-acuity complications related to TKA.

This study had several limitations. Since this was a retrospective database study, selection bias likely exists between study groups. We performed propensity score matching to limit potential confounders between patients, but since the dataset was completely de-identified, we were unable to control for the hospital the surgery was performed at. This likely influenced our results and explains the paradoxical trends in TA-TKA utilization within the dataset. This also likely influenced our analysis of short-term outcomes because there is significant heterogeneity between hospital systems in terms of demographics, patients’ social support, perioperative rehabilitation protocols, clinical care coordination, postoperative inpatient care, and access to multidisciplinary home service. All of these factors have been shown to reduce short-term complications and readmission rates [34–36]. Since the NSQIP database utilized CPT codes to record operative details, we were also unable to differentiate between computer navigation and robotic TKA.

Furthermore, NSQIP lacks specific ICD coding for readmission and reoperation diagnoses and does not record emergency department visits in which the patient is discharged. This likely caused our study to underrepresent the true complication rates, specifically superficial site infections in which the patient can receive antibiotics and then undergo follow-up with their primary care provider. Additionally, although technology use likely increased from 2018 to 2021, we were unable to capture this because, at the time of this analysis, the latest year of released NSQIP data was 2018. We also did not assess which patient characteristics are associated with technology utilization, though prior studies have investigated this issue [16, 19]. The NSQIP database only provided 30-day follow-up, which does not capture the true complication rate within the 90-day bundled period. Lastly, since our study was highly powered, our analysis detected miniscule differences as statistically significant. Given this, although certain clinical outcomes differed significantly, these differences may not be clinically significant.

## Conclusion

Our assessment of TA-TKA using the NSQIP database both aligns with and differs from that of previously reported TA-TKA patient demographic characteristics and utilization rates. This is the largest study to report operative times for TA-TKA, revealing that TA-TKA and U-TKA had similar operative times. Compared with U-TKA, patients undergoing TA-TKA were less likely to receive postoperative blood transfusions, had shorter mean LOS, and were more likely to be discharged home. Readmission rates for TA-TKA trended lower than U-TKA, though rates did not significantly differ between groups. The differences in perioperative outcomes may be explained by differences in providers and hospitals that perform TA-TKA as compared with those who only perform U-TKA, rather than implicit differences in surgical technique between the two modalities. Future research should examine how additional factors, such as hospital environment and geographical region, influence whether a patient receives TA-TKA over U-TKA to assess their impact on patient outcomes and technology utilization. Moreover, as national databases release data from more recent years, additional analyses should be performed to characterize how utilization trends evolve with time.

## Data Availability

The data that support the findings of this study are available from the American College of Surgeons National Surgical Quality Improvement Program database, but restrictions apply to the availability of these data, which were used under license for the current study, and so are not publicly available. Data are, however, available from the authors upon reasonable request and with permission of American College of Surgeons.

## Abbreviations

TKA: Total knee arthroplasty
TA-TKA: Technology-assisted total knee arthroplasty
U-TKA: Unassisted total knee arthroplasty
NSQIP: American College of Surgeons National Surgical Quality Improvement Program

## References

[1] Singh JA, Yu S, Chen L, Cleveland JD (2019). Rates of total joint replacement in the United States: future projections to 2020–2040 using the national inpatient sample. J Rheumatol.

[2] Freudenberger DC, Baker EA, Siljander MP, Rohde RS (2018). Factors driving patient perception of quality care after primary total hip and total knee arthroplasty. J Am Acad Orthop Surg Glob Res Rev..

[3] Rosenberger RE, Hoser C, Quirbach S, Attal R, Hennerbichler A, Fink C (2008). Improved accuracy of component alignment with the implementation of image-free navigation in total knee arthroplasty. Knee Surg Sports Traumatol Arthrosc.

[4] Haaker RG, Stockheim M, Kamp M, Proff G, Breitenfelder J, Ottersbach A (2005). Computer-assisted navigation increases precision of component placement in total knee arthroplasty. Clin Orthop Relat Res.

[5] Gausden EB, Popper JE, Sculco PK, Rush B (2020). Computerized navigation for total hip arthroplasty is associated with lower complications and ninety-day readmissions: a nationwide linked analysis. Int Orthop.

[6] Ren Y, Cao S, Wu J, Weng X, Feng B (2019). Efficacy and reliability of active robotic-assisted total knee arthroplasty compared with conventional total knee arthroplasty: a systematic review and meta-analysis. Postgrad Med J.

[7] Kim YH, Yoon SH, Park JW (2020). Does robotic-assisted TKA result in better outcome scores or long-term survivorship than conventional TKA? A randomized, controlled trial. Clin Orthop Relat Res.

[8] Aoude AA, Aldebeyan SA, Nooh A, Weber MH, Tanzer M (2016). Thirty-day complications of conventional and computer-assisted total knee and total hip arthroplasty: analysis of 103,855 patients in the American College of Surgeons National Surgical Quality Improvement Program Database. J Arthroplasty.

[9] Shaw JH, Lindsay-Rivera KG, Buckley PJ, Weir RM, Banka TR, Davis JJ (2021). Minimal clinically important difference in robotic-assisted total knee arthroplasty versus standard manual total knee arthroplasty. J Arthroplasty.

[10] Webb ML, Hutchison CE, Sloan M, Scanlon CM, Lee GC, Sheth NP (2021). Reduced postoperative morbidity in computer-navigated total knee arthroplasty: a retrospective comparison of 225,123 cases. Knee.

[11] Mavrogenis AF, Savvidou OD, Mimidis G, Papanastasiou J, Koulalis D, Demertzis N (2013). Computer-assisted navigation in orthopedic surgery. Orthopedics.

[12] Zheng G, Nolte LP (2015). Computer-assisted orthopedic surgery: current state and future perspective. Front Surg.

[13] Kamalapathy P, Hines J, Cui Q (2021). Navigation assisted total knee arthroplasty in 54,114 patients: no increased risk in acute complications and hospital utilisation. Int J Med Robotics Comput Assisted Surg.

[14] Ahmed AG, Kang R, Hasan M, Tian Y, Ghomrawi HM (2020). Trends in practice patterns of conventional and computer-assisted knee arthroplasty: an analysis of 570,671 knee arthroplasties between 2010 and 2017. J Am Acad Orthopaed Surg..

[15] Hussain A, Malik A, Halim MU, Ali AM (2014). The use of robotics in surgery: a review. Int J Clin Pract.

[16] Boylan M, Suchman K, Vigdorchik J, Slover J, Bosco J (2018). Technology-assisted hip and knee arthroplasties: an analysis of utilization trends. J Arthroplasty.

[17] Sodhi N, Khlopas A, Piuzzi NS, Sultan AA, Marchand RC, Malkani AL (2018). The learning curve associated with robotic total knee arthroplasty. J Knee Surg.

[18] Sherman WF, Wu VJ (2020). Robotic surgery in total joint arthroplasty: a survey of the AAHKS membership to understand the utilization, motivations, and perceptions of total joint surgeons. J Arthroplasty.

[19] Antonios JK, Korber S, Sivasundaram L, Mayfield C, Kang HP, Oakes DA (2019). Trends in computer navigation and robotic assistance for total knee arthroplasty in the United States: an analysis of patient and hospital factors. Arthroplasty Today.

[20] Naziri Q, Burekhovich SA, Mixa PJ, Pivec R, Newman JM, Shah NV (2019). The trends in robotic-assisted knee arthroplasty: a statewide database study. J Orthop.

[21] Shiloach M, Frencher SK, Steeger JE, Rowell KS, Bartzokis K, Tomeh MG (2010). Toward robust information: data quality and inter-rater reliability in the American College of Surgeons National Surgical Quality Improvement Program. J Am College Surg.

[22] Kane LT, Fang T, Galetta MS, Goyal DKC, Nicholson KJ, Kepler CK (2020). Propensity score matching. Clin Spine Surg.

[23] Caliendo M, Kopeinig S (2008). Some practical guidance for the implementation of propensity score matching. J Econ Survey.

[24] Austin PC (2009). Some methods of propensity-score matching had superior performance to others: results of an empirical investigation and Monte Carlo simulations. Biometrical J.

[25] Sekimura TK, Upfill-Brown A, Hsiue PP, Khoshbin A, Zeegen EN, Stavrakis AI (2021). Trends in operative time and short-term outcomes after conventional and navigated total knee arthroplasty. Arthroplasty Today.

[26] Turner M, Adam MA, Sun Z, Kim J, Ezekian B, Yerokun B (2017). Insurance status, not race, is associated with use of minimally invasive surgical approach for rectal cancer. Ann Surg.

[27] Gholson JJ, Duchman KR, Otero JE, Pugely AJ, Gao Y, Callaghan JJ (2017). Computer navigated total knee arthroplasty: rates of adoption and early complications. J Arthroplasty.

[28] Liodakis E, Antoniou J, Zukor DJ, Huk OL, Epure LM, Bergeron SG (2016). Navigated vs conventional total knee arthroplasty: is there a difference in the rate of respiratory complications and transfusions?. J Arthroplasty.

[29] Ofa SA, Ross BJ, Flick TR, Patel AH, Sherman WF (2020). Robotic total knee arthroplasty vs conventional total knee arthroplasty: a nationwide database study. Arthroplasty Today.

[30] Onggo JR, Onggo JD, de Steiger R, Hau R (2020). Robotic-assisted total knee arthroplasty is comparable to conventional total knee arthroplasty: a meta-analysis and systematic review. Arch Orthop Trauma Surg.

[31] Klika AK, Small TJ, Saleh A, Szubski CR, Chandran Pillai ALP, Barsoum WK (2014). Primary total knee arthroplasty allogenic transfusion trends, length of stay, and complications: nationwide inpatient sample 2000–2009. J Arthroplasty.

[32] Kayani B, Konan S, Pietrzak JRT, Haddad FS (2018). Iatrogenic bone and soft tissue trauma in robotic-arm assisted total knee arthroplasty compared with conventional jig-based total knee arthroplasty: a prospective cohort study and validation of a new classification system. J Arthroplasty.

[33] Keswani A, Tasi MC, Fields A, Lovy AJ, Moucha CS, Bozic KJ (2016). Discharge destination after total joint arthroplasty: an analysis of postdischarge outcomes, placement risk factors, and recent trends. J Arthroplasty.

[34] Peters CL, Shirley B, Erickson J (2006). The effect of a new multimodal perioperative anesthetic regimen on postoperative pain, side effects, rehabilitation, and length of hospital stay after total joint arthroplasty. J Arthroplasty.

[35] Losina E, Walensky RP, Kessler CL, Emrani PS, Reichmann WM, Wright EA (2009). Cost-effectiveness of total knee arthroplasty in the United States patient risk and hospital volume. Arch Intern Med.

[36] Fitzgerald SJ, Palmer TC, Kraay MJ (2018). Improved perioperative care of elective joint replacement patients: the impact of an orthopedic perioperative hospitalist. J Arthroplasty.

